# Alcohol’s Role in Work-Force Entry and Retirement

**Published:** 1996

**Authors:** Paul M. Roman, J. Aaron Johnson

**Affiliations:** Paul M. Roman, Ph.D., is director and J. Aaron Johnson, M.A., is a predoctoral trainee at the Center for Research on Deviance and Behavioral Health, Institute for Behavioral Research, University of Georgia, Athens, Georgia

**Keywords:** employment, life event, young adult, adolescent, elderly, heavy AOD use, AOD use behavior, coping skills, aging, social role, AOD tolerance, psychological stress, leisure activity

## Abstract

Employment and drinking behavior interact in intriguing ways both upon entrance to and departure from the labor force. Teenagers who work are more likely to drink than their unemployed peers, possibly offsetting the expected advantages of gaining early job experience. For young people in general, early heavy drinking can curtail continuing education and stifle opportunities for career advancement. At the opposite end of the age spectrum, some retirees may turn to alcohol to fill leisure time and cope with the stresses associated with retirement as a major life change. Other retirees, however, may cut back on drinking once they are freed from job-related stress, leave a work environment that encourages alcohol use, or experience financial constraints. Although tolerance to alcohol’s effects wanes with advancing age, older adults who remain employed are more apt than retirees to drink heavily. Alternatively, older workers may decide to restrict their drinking to keep pace with younger colleagues.

The study of drinking behavior as it relates to work roles over a person’s life span offers researchers exciting possibilities as well as special challenges. Most alcohol-related literature is based on data measured at a single point in time (i.e., cross-sectional data). As a result, this research inadvertently presents a static portrait of the links between alcohol problems and psychosocial characteristics, such as depression and stress. For a more complete picture, studies must be designed to follow the lives and drinking careers of people who have work-related psychosocial experiences that may be damaging or deadly relative to alcohol use. For example, it would be valuable to study how drinking behavior interacts with employment in occupations that have a high potential for competitive stress, failure, or downward mobility from the “pinnacles of success.” Although no in-depth examination of drinking behavior across occupational careers has been undertaken to date, alcohol researchers are reasonably confident that work and alcohol consumption can be linked on a variety of levels (Roman 1990; Vaillant 1995).

This article describes recent research on employment and drinking at selected life stages. A discussion covering the full breadth of data on drinking and various adult life passages would be well beyond the article’s space limitations. Moreover, because of the difficulty in isolating the study of “prime-age” drinking behavior from the entire body of research on normal and deviant drinking behavior, this article narrows its focus to examine two particular groups of people: those entering and those leaving employment roles.

## Three General Observations

As time passes during adulthood, three noted phenomena can impact drinking behavior: (1) drinking problems^1^ frequently are resolved without serious crises or outside assistance, (2) tolerance for alcohol’s effects decreases, and (3) social roles tend to diminish. Although these phenomena have intriguing theoretical implications, their interaction with employment remains largely unexplored to date.

### Simple Resolution of Drinking Problems

Epidemiological data clearly show that a substantial proportion of drinking problems apparently are resolved without major crises, interventions, or treatment (i.e., the problems resolve themselves by “spontaneous remission”) (Tuchfeld 1981). Data also indicate that 15.4 percent of Americans who consume alcohol have a drinking problem^1^ at some point in their lives and that these problems primarily occur during adulthood (Robins and Regier 1990). Given that work roles are central to the lives of many, if not most, adult Americans, both the origin and diminution of alcohol problems are likely to be work related for some people. Conversely, as Mullahy and Sindelar (1989, 1991, 1994) so effectively argue in their research, alcohol problems may significantly affect a person’s occupational opportunities. The following discussion addresses both views of the alcohol-work relationship.

In an early observation, Roman and Trice (1970) noted a probable association between job changes and either the alleviation or exacerbation of alcohol problems. For example, a person with an incipient drinking problem in a high-stress occupation may seek a less stressful job as a means of alleviating both dilemmas. Conversely, some people may seek employment changes that enhance their opportunities to drink. Ames and Janes’ (1990, 1992) research in the area of occupational drinking subcultures suggests that certain occupations are more conducive to and accepting of workplace drinking (e.g., jobs in which stress levels are high, tasks are repetitious and boring, or work is performed alone or under little supervision). Given this tolerance of drinking found in some work cultures, it seems likely that people with alcohol problems might seek occupations in which drinking behavior is relatively easy to hide or in which coworkers promote (or at least accept) deviant drinking behavior (Roman et al. 1992). Practically nothing is known about these dynamics, although they clearly exist by inference.

### Effects of the Aging Process

The process of aging is strongly associated with what appears to be the alleviation or resolution of drinking problems (Mirand and Welte 1996). Many people apparently “age out” of patterns of excessive or problematic drinking, and this occurrence often is attributed to a decreased tolerance for alcohol as a result of the aging process. Frequently, older adults experience a compromised ability to cope with alcohol’s effects for physiological reasons, such as a lower water content in the body and a reduced lean body mass (Finney and Moos 1984; Mertens et al. 1996). In addition, the health and medical problems that often plague older adults may be aggravated by even moderate levels of alcohol consumption. Such decreased physiological ability to handle the effects of drinking reflects an Alcoholics Anonymous (AA) expression that associates the decision to seek sobriety with “being sick and tired of being sick and tired.” This phrase clearly applies to a broader population than AA participants alone, however.

Along with the physiological aspects of aging, psychosocial elements may be equally important influences on drinking behavior and may have a direct effect on work performance. Older workers, keenly aware of their need to “keep up,” may intentionally make lifestyle changes, such as reducing or quitting drinking, in an effort to maintain skill and productivity on the job (Roman 1990). Such responses probably vary, though, depending on the competitiveness of the job circumstances as well as the scope of alternative opportunities.

**Figure:**
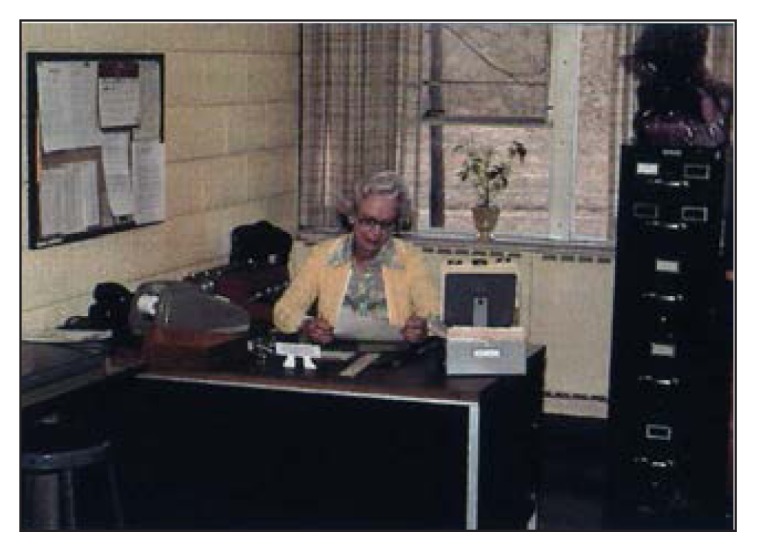


### Diminished Social Roles

Besides spontaneous remission and decreased alcohol tolerance, the third phenomenon of interest is related to the concept of “role shrinkage,” an idea developed by an industrial clinical psychologist specializing in treatment of executives with alcohol problems (P.A. Sherman, personal communication, April 1996). The idea is based on the once-controversial sociological concept of “disengagement” developed in social gerontology (Cumming and Henry 1961). According to this theory, a person narrows the scope of his or her social participation with advancing age. Society’s social expectations for this age group also usually decline and are evidenced by retirement, the “empty nest” syndrome, and age-segregated residential patterns. Disengaging from life roles related to work or parenting, for example, may be both socially expected and physiologically determined, but gerontologists tend to find such disengagement socially defeating for older people, who may become withdrawn and isolated as a result (Reitzes et al. 1995).

Although not yet fully documented, excessive drinkers (even those considerably younger than retirement age) may deliberately choose role shrinkage. Through a pattern of “false disengagement,” they may restrict their activities to a smaller number of tasks that they can continue to perform well in an attempt to cover up for excessive, deviant, or damaging drinking behavior. This theory is based on the premise that alcohol use can adversely affect job performance, which in turn can make problematic drinking behavior visible, especially in interaction with the aging process.

Despite patterns of excessive drinking, some people may be able to maintain excellence in work-role performance for an extended period (Knapp 1996). Eventually, however, these patterns exact a toll and motivate the person toward role shrinkage. In such cases, changes in work performance may be subtle, making it difficult for observers to detect any patterns of deterioration. More important, researchers must endeavor to distinguish alcohol-associated effects, which are potentially reversible, from age-associated ones, which are less likely to change.

## Assessing the Relationship Between Alcohol and Work at Different Life Stages

To integrate the findings discussed in the following sections, data from two prominent national surveys were analyzed. First, the National Household Survey on Drug Abuse (NHSDA) was used to examine the youthful end of the age spectrum (see table 1). The National Institute on Drug Abuse conducts this survey annually to measure the prevalence and correlates of drug use in the United States with a nationally representative sample. The NHSDA gathers information on the use of illicit drugs, alcohol, and tobacco from members of the U.S. household population ages 12 and older.^2^

Second, the Americans’ Changing Lives (ACL) survey, conducted by the Survey Research Center of the University of Michigan, was used to consider patterns among older adults (see table 2). The ACL gathered information on interpersonal relationships, life satisfaction, perceptions of retirement, health behaviors, and so forth from a nationally representative sample of adults ages 25 and older.^3^

To assess the drinking patterns in these two surveys, a scale was constructed similar to that developed by Cahalan and Cisin (1968).^4^ Respondents were classified into one of five general quantity-frequency drinking groups: abstainers, infrequent drinkers, light drinkers, moderate drinkers, and heavy drinkers. Abstainers were those who consumed alcohol less than once per year or not at all. Infrequent drinkers were those who had not had a drink containing alcohol in the past 30 days. Light drinkers consumed alcohol at least once per month, typically with no more than one or two drinks on any occasion. Moderate drinkers consumed alcohol at least once per month (typically several times per month), with no more than three or four drinks per drinking occasion. Heavy drinkers consumed alcohol nearly every day, with five or more drinks in a single sitting at least occasionally, or they consumed alcohol at least weekly, with five or more drinks on most drinking occasions.^5^

Data from these two surveys supplement the findings of other researchers discussed in the following sections.

## Alcohol, Employment, and Teenagers and Young Adults

Although it is illegal for high school and nearly all college students (i.e., those younger than age 21) to purchase and consume alcoholic beverages, research indicates that almost everyone in this age group reports some experience with alcohol (Johnston et al. 1992). Furthermore, approximately 30 percent of high school seniors and 43 percent of college students report occasions of heavy drinking^6^ (Johnston et al. 1992). The potential impact of alcohol abuse on careers is perhaps greatest for teenagers and young adults, because alcohol problems developing at these young ages can adversely affect the person’s future work for years to come (Mullahy and Sindelar 1994). Mullahy and Sindelar (1994, 1989) found that people experiencing alcohol problems at age 18 or younger generally were less educated, less likely to have white-collar jobs, and more likely to have lower incomes than their counterparts not experiencing alcohol problems. The reduced likelihood of having a white-collar job was even more pronounced when the onset of alcohol problems occurred between the ages of 19 and 22 (Mullahy and Sindelar 1989). These findings were based on data from respondents ages 25 to 64, an age range deliberately chosen so that the outcomes would reflect the responses of subjects most likely to have completed their education yet not likely to have retired from the labor force.

In searching for variables that could predict the likelihood of a young adult’s being in the work force or in school, Sanford and colleagues (1994) found that the strongest predictors of being either out of school or unemployed were low family income and heavy substance use. Of the four groups in the study (i.e., attending school, employed full time, employed part time, and unemployed), the unemployed group was found to have the highest rates of substance use and other predictor variables, such as low family income, low maternal education, and remedial education. Like Mullahy and Sindelar, Sanford and colleagues (1994) argued that the early onset of alcohol and other drug abuse “propel[s] a child along pathways toward negative work force outcome” (p. 1044).

Interestingly, Mullahy and Sindelar (1989, 1992) found that young adults with alcohol problems initially appeared to earn higher wages than young adults without drinking problems. This discrepancy may reflect Sanford and colleagues’ (1994) finding that nonalcoholics in this age group were more likely to be in school. Thus, young adults with alcohol problems may have more experience in the labor market and work more hours than their counterparts without drinking problems (Mullahy and Sindelar 1992). However, as time passes and those without drinking problems leave school and gain work experience, the nonalcoholics’ salaries are likely to catch up to and surpass the income of young adults with alcohol problems.

Mullahy and Sindelar (1992) note that the higher incomes found initially among the problem-drinking young adults do not imply that these young adults were better off than their age peers without alcohol problems. Given what is known about job-associated educational requirements, many of these young problem drinkers clearly were in occupations that offered few long-term rewards and virtually no chance for advancement (e.g., assembly-line, construction, or sanitation work). Occupations such as these are attractive, however, because of their immediate rewards, and significant time may pass before workers in these jobs realize the “downsides” of the occupation.

Any discussion of alcohol’s role in the career potential of young adults cannot ignore the vast research attention given to drinking among college students. Researchers repeatedly have replicated findings that college students tend to binge drink, drink heavily, and drink dangerously. Furthermore, data show that college students drink more than their age peers who are not attending college (Johnston et al. 1992). A host of additional variables must be considered when analyzing collegiate drinking and when forming predictions of whether these patterns will persist through the postcollege years; for example, the affiliations students acquire while attending college may be important influences. Despite the adverse consequences associated with drinking by college students, most students who drink will graduate and, consequently, reap the job-market rewards and advantages associated with a college degree.

The effects of teen employment on alcohol use and abuse constitute a different set of issues. The importance of the work ethic in American culture tends to encourage youthful employment and behaviors that may be seen as precursors for adult careers. However, employment is strongly correlated with alcohol use and abuse among teenagers (Steinberg et al. 1993; Steinberg and Dornbusch 1991). Steinberg and colleagues (1993) found that previously unemployed teenagers who were employed at the 1-year followup were using alcohol and other drugs (AOD’s) significantly more often than their counterparts who had remained unemployed. Furthermore, as the number of work hours increased, teenagers reported higher rates of AOD use (Steinberg and Dornbusch 1991). Steinberg and colleagues (1993) also found that employed teenagers were more likely to report patterns of heavy alcohol consumption. These findings complement the research of Sanford and colleagues (1994) and Mullahy and Sindelar (1989, 1991) discussed previously.

Taking these findings one step further, employed teenagers who subsequently experience the early onset of drinking problems may be less likely to continue their education beyond high school. This implication seems to be supported by the research on young adults conducted by both Sanford and colleagues (1994) and Mullahy and Sindelar (1989, 1991). Sanford and colleagues (1994) found that young people engaged in heavy substance use were significantly less likely to be in school; similarly, Mullahy and Sindelar (1989, 1991) found that early onset of alcohol problems was correlated with lower levels of education. Furthermore, as the findings of Sanford and colleagues (1994) appear to support, teenagers who experience an early onset of drinking problems may find themselves unemployed as young adults if they have been unable to control their drinking. Unfortunately, confirmation of these implications would require the use of longitudinal data following both employed and unemployed teenagers for at least a decade, and such data do not exist.

Although none of the cited findings on the alcohol-employment relationship among young people is based on data from a nationally representative sample, our analysis of the 1988 NHSDA data provides some verification on a national level. As table 1 illustrates, 15- to 17-year-old employed males (*p* < 0.01) and females (*p* < 0.001) are significantly more likely to be classified as heavy or moderate drinkers than their unemployed counterparts. In contrast, unemployed male and female teenagers are more likely to report being abstainers.

These findings appear to contradict the popular conception that teen employment outside the home instills discipline and responsibility. Instead, these findings suggest that employed teenagers are more highly involved in AOD use than their unemployed peers and that such involvement seems to increase as work hours increase. Steinberg and Dornbusch (1991) offer three possible explanations for this discrepancy: (1) workers have more discretionary income to use for purchasing alcohol, (2) workers experience more stress than nonworkers and consequently turn to alcohol as a means of coping with this additional stress, and (3) workers encounter older adolescents and young adults in the workplace who expose them to drinking activities.

Unfortunately, the majority of studies in this area, including the research conducted by Mullahy and Sindelar, have relied on cross-sectional data and, as such, provide a limited picture of the alcohol-employment relationship. Consequently, it is difficult to specify a causal direction for the alcohol-employment relationship among teenagers and young adults. It seems logical, however, that the relationship could be bidirectional. Based on any of the reasons suggested by Steinberg and Dornbusch, teen employment could lead to increases in alcohol consumption. In turn, those teenagers and young adults who engage in heavy alcohol use or abuse may be less likely to either continue in school or obtain a white-collar job.

As a result, some of these young problem drinkers may become locked into the lower class, a hypothesis supported by Vaillant (1995). His longitudinal research found that although most lower class boys could improve their socioeconomic status as adults, those boys with low intelligence, psychiatric disorders, alcohol dependence, or a combination of such factors were found in the lower class as adults regardless of their original socioeconomic background. (For further information, see the article by Vaillant, beginning on p. 152.)

## Alcohol, Employment, and Older Adults

As American society prepares to enter the new millennium, issues relating to older adults continue to gain increasing prominence. Current projections estimate a dramatic increase in the number of Americans over age 65 in the coming decades, from 39.4 million in 2010 (13.2 percent of the total population) to 69.4 million in 2030 (20 percent of the total population) (Day 1996). In light of the aging population, debate over medicare undoubtedly will be a major political issue well into the 21st century, perhaps leading to heightened tensions between different age groups in the population. With the health care situation of millions of people possibly in the balance, it is more crucial than ever to explore the extent and causes of alcohol abuse among the older American population.

In considering the effects of alcohol use and abuse among older adults, several inherent factors contribute to an overall decrease in alcohol consumption among this population. First, adults age 60 or older are unlikely to be heavy drinkers, simply because the heaviest drinkers tend to succumb at an earlier age from physical complications, accidents, or other injuries related to excessive alcohol consumption (Mirand and Welte 1996; Mertens et al. 1996). Also, as previously discussed, the aging process results in decreased physical tolerance for alcohol’s effects during and after drinking episodes, leading to reduced consumption (also see the article by Brennan, beginning on p. 197).

Much speculation but sparse facts exist regarding the effects of occupational retirement on alcohol consumption. For many people, retirement may be a particularly stressful period in which they experience a loss of status, boredom, depression, loss of self-esteem, or general discordance as to what their roles are as members of society (Ekerdt et al. 1989). Researchers conjecture that retirees turn to alcohol to help them cope with this stress (Ekerdt et al. 1989; Finney and Moos 1984). In addition, researchers speculate that older men may be more at risk for such stress-related alcohol abuse than older women, because men, particularly the current generation of retirees, tend to be more highly integrated into the labor force and are more likely to hold high-status managerial jobs. At retirement, this status loss may be especially difficult for men to accept.

In addition to increased stress, the retiree has more leisure time in which to consume alcohol. With few role constraints or social obligations, the retiree may consume alcohol with greatly reduced risks of adverse social consequences (Ekerdt et al. 1989). Because men report higher levels of alcohol consumption than women throughout life (Cahalan and Cisin 1968), leisure-related alcohol consumption associated with retirement also may occur more frequently among men than women.

Living arrangements also may influence the drinking behavior of retirees. A survey conducted by Alexander and Duff (1988) revealed that drinking was more common among residents of retirement communities than among their age peers in the general population. Furthermore, a strong relationship was found between alcohol use and greater social interaction. Widespread social drinking appeared to be an integral part of the leisure subculture in these communities; several survey respondents even said that residents feel they must drink to be accepted in some community groups (Alexander and Duff 1988).

In the Normative Aging Study, Ekerdt and colleagues (1989) attempted to address the effects of retirement on alcohol consumption systematically by assessing pre- and postretirement changes in male retirees’ drinking behaviors. The group of retirees was then compared with a similar group of age peers who continued to work. Although the study data were based on a small sample size and a single geographical location, Ekerdt and colleagues (1989) found that retirement was not associated with a change in average alcohol consumption. They did find, however, that retirees showed greater variability over time in consumption levels: A number of retirees reported heavy drinking patterns, whereas others reported the cessation of heavy drinking and its related problems. Finally, retirees were more likely than their employed age peers to report the onset of periodic heavier drinking and drinking problems.

In contrast, other researchers suggest that a significant decrease occurs in the level of alcohol consumption as people approach retirement age (Barnes 1979; Gurnack and Thomas 1989). This decline may be attributed to a number of factors. Retirement may eliminate the job stress that can be correlated with heavy drinking; retirement also may remove people from workplace subcultures in which heavy drinking had been not only acceptable but encouraged. Ames and Janes (1990) found that some work-place environments and work-related social networks serve to develop and maintain heavy-drinking practices. After leaving such an environment, retirees may reduce their alcohol consumption levels substantially. For many older adults, another possible contributor to the decline in alcohol consumption is reduced income during retirement, which financially may limit their access to alcoholic beverages.

**Figure:**
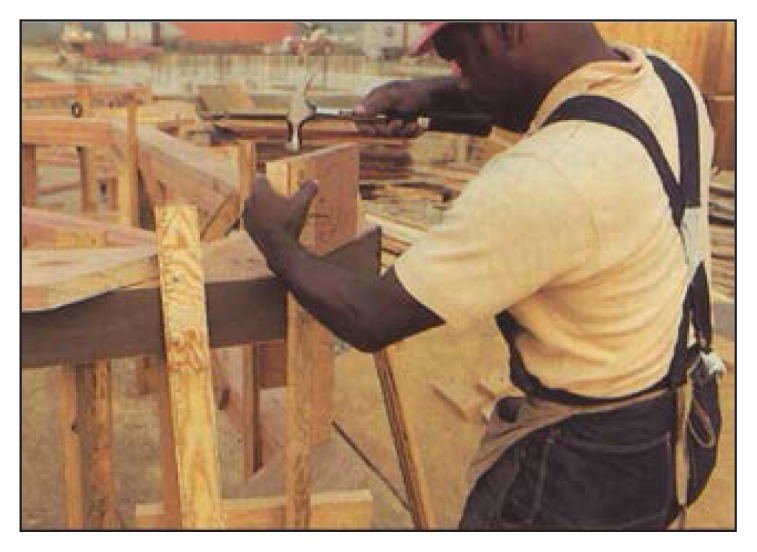


Barnes (1979) strongly argues that retirement is not related to an increase in heavy drinking. She found that adults over age 60 who were still employed were twice as likely to be heavy drinkers as those who were unemployed or retired. This finding is supported by Mullahy and Sindelar (1991), who observed that alcohol abusers tended to remain in the labor force longer than nonalcoholics. The researchers reasoned that workers without alcohol problems are able to accrue greater wealth and larger pensions than alcoholics and therefore may retire, whereas alcoholics must continue to work.

The data in table 2 further support Barnes’ (1979) findings. Although Barnes (1979) found that among the younger workers (i.e., those ages 18 to 49 and 50 to 59), the unemployed subjects were significantly more likely to be heavy drinkers than their employed counterparts, the case for older adults was precisely the opposite. Likewise, table 2 shows that the employed males (*p* < 0.001) and females (*p* < 0.05) over age 60 were significantly more likely than their unemployed (i.e., retired) peers to be classified as heavy or moderate drinkers, whereas the unemployed males and females were more likely to be classified as abstainers. Although only replicating Barnes’ study, these data and findings broaden its generalizability with a nationally representative sample.

In summary, stress and additional leisure time associated with retirement may adversely affect some older people by increasing their alcohol consumption to an abusive level, but the extent of such a pattern may not be large. Only Ekerdt and colleagues (1989) and Alexander and Duff (1988) found that retirees are likely to experience the onset of periodic heavy drinking. Gurnack and Hoffman (1992) attribute abusive drinking among retirees to a history of alcohol abuse, not to age-related stress. Barnes’ (1979) research, as well as our own analysis, shows that employed older adults, rather than retirees, are more likely to report high levels of alcohol consumption.

Most of the current literature suffers, however, from the following basic weaknesses: a small sample size, a single geographic location, or both (Gurnack and Thomas 1989). Furthermore, the majority of these studies only speculate as to the causes of alcohol abuse among older adults rather than provide data-based analyses. Because of the limitations of the data in these studies, it is therefore premature to conclude that a relationship does not exist between retirement and alcohol abuse among the older adult population.

## Conclusion

This brief foray into the research literature demonstrates the potential richness of the three-way connections among drinking, employment, and the life cycle. From a public policy perspective, it is disturbing that employment among teenagers may be adversely associated both with drinking behavior and with other behaviors that can hinder positive growth during late adolescence. A heritage of successful social activism protecting children from abuses in the workplace segregates youth in American society from participation in work roles. However, opportunities for adolescent involvement in the work force are viewed positively and are culturally encouraged. Thus, it is additionally troubling to find that these opportunities are not necessarily a gateway to upward occupational mobility over the life span as expected but, in fact, potentially the opposite. From the available data, it appears possible that experiences in at least some teen employment settings (e.g., fast food or retail settings) may set a persistent pattern that limits opportunities for growth and advancement. Further research is needed, however, to address the reasons why teenagers enter employment and how these reasons may put them at risk for potential problems with alcohol.

Findings about the job choices of young adults who are problem drinkers offer broad illustrations of the intermingling of life course, employment, and drinking variables. Frequently, the dead-end jobs in which these employees find themselves are highly routinized and have minimal cognitive demands. Consequently, excessive drinking off the job or even on the job may not interfere with work performance. In addition, because youthful workers are better able to compensate for the adverse effects of hangovers than are their older coworkers, blocked career mobility may not be realized until much later.

Generalizations about drinking among retirement-age adults are considerably more difficult. It is likely that much depends on the nature of the job, its fit with the person’s physical capabilities, and the person’s motivation to continue working. For many reasons, retirement is a loss and disappointment for some workers, whereas it is a release and a reward for others. Likewise, work settings vary greatly in their treatment of older workers: In some workplaces, older employees are considered wise mentors, but in other situations, they may be viewed as holding choice positions deserved by younger employees.

As “baby boomers” approach retirement age, their drinking behavior during the later employment and retirement years could assume considerable policy significance. For example, if freedom to enjoy an economically secure retirement is curbed (e.g., because drinking behavior has locked a worker into a low-paying, dead-end job), older adults may delay retirement to continue earning an income. Such a scenario might trigger resentment among younger employees who expect advancement opportunities when their older coworkers retire. Meanwhile, to confirm that they are indeed capable of continued job performance, employed older drinkers may reduce their alcohol consumption and contribute to an overall decline in drinking among older adults. Conversely, retirees who are segregated from the working population and equipped with adequate economic resources actually may facilitate the growth of drinking-oriented subcultures if society fails to promote widely available activities that are meaningful alternatives to work.

## Figures and Tables

**Table 1:** Drinking Classifications According to Age, Sex, and Employment Status (15- to 17-Year-Olds)

	*N*	Drinking Classification (%)				

		Heavy	Moderate	Light	Infrequent	Abstainer
**Employed**						
Males^1^	163	6.1	18.4	11.7	28.8	35.0
Females^2^	173	1.2	11.0	29.5	30.6	27.7
**Unemployed**						
Males^1^	516	4.1	8.5	12.6	29.7	45.2
Females^2^	513	0.4	5.1	15.8	30.8	48.0

1 *p* < 0.01.

2 *p* < 0.001.

SOURCE: 1988 National Household Survey on Drug Abuse.

**Table 2:** Drinking Classifications According to Age, Sex, and Employment Status (Age 60 and Older)

	*N*	Drinking Classification (%)				

		Heavy	Moderate	Light	Infrequent	Abstainer
**Retirees**						
Males^1^	175	5.1	10.3	25.1	50.3	9.1
Females^2^	183	1.1	2.7	20.8	42.6	32.8
**Employed**						
Males^1^	50	8.0	14.0	22.0	38.0	18.0
Females^2^	57	0.0	5.3	22.8	36.8	35.1

1 *p* < 0.001.

2 *p* < 0.05.

SOURCE: Americans’ Changing Lives: Wave 1, 1986.

## Footnotes

1 The exact definition of terms such as “drinking problems,” “alcoholism,” and “alcohol abuse” can vary considerably across studies. Readers are encouraged to consult the original source for precise definitions as appropriate.

2 To improve the validity of the results, a confidential, anonymous questionnaire is administered within each respondent’s household. For drug-related and other potentially threatening questions, the respondent is allowed to use self-enumerated answer sheets. Each NHSDA includes an oversample of young people, minorities, and the Washington, DC, Metropolitan Statistical Area. Our analyses are based on the 1988 NHSDA, which contained a sample size of 7,249 respondents.

3 The ACL survey includes an oversample of blacks and adults age 60 and older. In 1986 a total of 3,617 respondents were interviewed in person. The response rate was 70 percent; non-response did not vary substantially by age, race, or other sociodemographic characteristics.

4 The ACL and NHSDA contain identical questions for measuring the frequency and quantity of alcohol use among respondents. The question measuring frequency asks: “During the past 30 days, on about how many different days did you have one or more drinks?” The question for assessing quantity asks: “On the days that you drank in the past 30 days, about how many drinks did you usually have in a day?”

5 These classifications are based on Cahalan and Cisin’s (1968) principle that people who consume larger amounts of alcoholic beverages when drinking should receive a heavier drinking classification than those drinking about the same volume but in small quantities over a longer period. Cahalan and Cisin (1968) argue that it is unlikely that those who do not drink as many as five drinks on any occasion would become intoxicated or have serious drinking-related problems.

6 Here, occasions of heavy drinking are measured by the percentage of respondents reporting five or more drinks in a row at least once in the prior 2-week period.
